# Direct Evidences for Sympathetic Hyperactivity and Baroreflex Impairment in Tako Tsubo Cardiopathy

**DOI:** 10.1371/journal.pone.0093278

**Published:** 2014-03-25

**Authors:** Angelica Vaccaro, Fabien Despas, Clement Delmas, Olivier Lairez, Elisabeth Lambert, Gavin Lambert, Marc Labrunee, Thibaut Guiraud, Murray Esler, Michel Galinier, Jean Michel Senard, Atul Pathak

**Affiliations:** 1 National Institute of Health and Medical Research (INSERM) UMR-1048, Institute of Metabolic and Cardiovascular diseases, Toulouse, France; 2 Toulouse University III Paul Sabatier, Toulouse, France; 3 Clinical Pharmacology Department, University Hospital of Toulouse, Toulouse, France; 4 Department of Cardiology, University Hospital of Toulouse, Toulouse, France; 5 Human Neurotransmitter Laboratory, Baker IDI Heart and Diabetes Institute and Faculty of Medicine, Nursing and Health Sciences, Monash University, Melbourne, Victoria, Australia; 6 C.I.C., Clinical Investigation Center, University Hospital of Toulouse, Toulouse, France; University of Adelaide, Australia

## Abstract

**Background:**

The exact pathophysiology of Tako-Tsubo cardiomyopathy (TTC) remains unknown but a role for sympathetic hyperactivity has been suggested. Up to now, no direct evidence of sympathetic nerve hyperactivity has been established nor involvement of sympathetic baroreflex identified. The aim of our study was to determine, by direct sympathetic nerve activity (SNS) recording if sympathetic nervous system activity is increased and spontaneous baroreflex control of sympathetic activity reduced in patients with TTC.

**Methods:**

We included 13 patients who presented with TTC and compared their SNS activity and spontaneous baroreflex control of sympathetic activity with that of 13 control patients with acutely decompensated chronic heart failure. SNS activity was evaluated by microneurography, a technique assessing muscle sympathetic nerve activity (MSNA). Spontaneous baroreflex control of sympathetic activity was evaluated as the absolute value of the slope of the regression line representing the relationship between spontaneous diastolic blood pressure values and concomitant SNS activity. Control patients were matched for age, sex, left ventricular ejection fraction and creatinine clearance.

**Results:**

The mean age of the patients with TTC was 80 years, all patients were women. There were no significant differences between the two groups of patients for blood pressure, heart rate or oxygen saturation level. TTC patients presented a significant increase in sympathetic nerve activity (MSNA median 63.3 bursts/min [interquartile range 61.3 to 66.0] vs median 55.7 bursts/min [interquartile range 51.0 to 61.7]; p = 0.0089) and a decrease in spontaneous baroreflex control of sympathetic activity compared to matched control patients (spontaneous baroreflex control of sympathetic activity median 0.7%burst/mmHg [interquartile range 0.4 to 1.9] vs median 2.4%burst/mmHg [interquartile range 1.8 to 2.9]; p = 0.005).

**Conclusions:**

We report for the first time, through direct measurement of sympathetic nerve activity, that patients with TTC exhibit elevated SNS activity associated with a decrease in spontaneous baroreflex control of sympathetic activity. These data may explain the pathophysiology and clinical presentation of patient with TTC.

## Introduction

Tako-Tsubo Cardiomyopathy (TTC) is an acute reversible condition characterized by left ventricular apical ‘ballooning’ and mimics acute myocardial infarction. It was first described in Japan in 1990 by Sato et al. [1] and the Japanese name ‘tako-tsubo’ describes the visual appearance of left ventricle on ventriculography resembling a fishing jar used to trap octopus. Since then, several cases have been described all over the world and TTC has been recognized as a primary acquired cardiomyopathy in the American Heart Association classification of cardiomyopathies [2].

Several studies have estimated that approximately 1% to 2% of all patients presenting with an initial primary diagnosis of acute coronary syndrome (ACS) have TTC [3]–[7]. TTC typically affects aged postmenopausal women [8], with less than 3% of patients being younger than 50 years [9], [10]. While TTC is usually triggered by a profound emotional or physical stress, in around 30% of cases no preceding stressful event could be identified [11]. The clinical presentation of TTC mimics ACS with ischemia-like chest pain and ischemia-like electrocardiographic changes contrasting with minimal elevation of cardiac enzymes despite the presence of large regions of focal myocardial akinesia involved [12], [13]. At coronary angiography there is a lack of identifiable obstructive coronary artery disease. Transient left apical and middle ventricular walls dysfunction with akinesia or dyskinesia (apical ‘ballooning’) is detectable. The abnormal ventricular ejection fraction observed during the acute phase rapidly improves over a period of days to weeks. The recurrence of TTC is infrequent: an average yearly recurrence rate of 2.9% over the first 4 years, subsequently decreasing to 1.3% per year [14]. Long-term survival and prognosis are debated. Previous studies reported that survival in TTC patients is similar to that expected for an age, gender matched population [14] while other studies showed that survival is significantly reduced [15] with a high rate of malignancies observed in TTC patients [16].

To date the pathogenesis of TTC remains uncertain. Sympathetic nervous system (SNS) activation is believed to contribute. Experimental and clinical data support the hypothesis that sympathetic hyperactivity may cause myocardial stunning and contractile dysfunction through catecholamine mediated mechanism [17]. This has been established through analysis of catecholamine levels obtained from circulating systemic sources or from cardiac sympathetic nerves (Norepinephrine (NE) spill over and ^123^I-métaiodobenzylguanidine (MIBG) myocardial scintigraphy) [18]–[20]. It has also been shown that exposure to catecholamines and beta-receptor agonists can precipitate stress cardiomyopathy [21]. Elevated catecholamine levels could impact directly on cardiac toxicity but also exert indirect effects on vessels to further injury the cardiovascular system. While the previous studies underline a potential role for elevated SNS activity some of them suffer from drawbacks such as small sample size, inappropriate controls (such as patients with myocardial infarction) or even absence of any control. The assessment of NE plasmatic levels is a technique with poor reproducibility and sensitivity being influenced by noradrenaline transporter function (NE reuptake and clearance from circulation) [22]. Given that as much as 80% of neuronally released NE in the heart is taken up by the neuronal NE transporter the heart is more susceptible to other organs to defects in norepinephrine transporter function [23]. Some studies used myocardial scintigraphy with ^123^I-MIBG [20] that is known to be less informative in patients with severe left ventricular dysfunction [24]. Other studies have attempted to demonstrate sympathetic hyperactivation using indirect SNS measurement techniques, such as heart rate variability [25], known to be influenced by other systems (i.e.: NO release, temperature, thermoregulation) and to not reflect cardiac sympathetic nerve activity [26] and cardiac NE spillover. Finally beyond these simple estimates of SNS activity, only one study has analyzed the status of sympathetic reflexes. In this single case report, TTC was associated with an acute disseminated encephalomyelitis affecting solitary tract nuclei where baroreceptors afferences converge [27].

We therefore decided to undertake the present study, to evaluate if TTC was associated with an increase in sympathetic nerve activity. This was assessed through direct muscle sympathetic nerve activity (MSNA) recording by microneurography, considered as the gold standard technique to assess central sympathetic drive to peripheral muscles. We also tested the hypothesis whether sympathetic baroreflex function is depressed in TTC patients contributing to the increase in SNS activity.

## Materials and Methods

To test our hypothesis we compared SNS activity, assessed by microneurography, and spontaneous baroreflex control of sympathetic activity, between a group of TTC patients and control patients being patient with acutely decompensated chronic heart failure.

### Patients

Between January 2010 and March 2011, 13 patients who fulfilled the Mayo Clinic Criteria [28] for the diagnosis of TTC were identified from the patients admitted at Toulouse University Hospital for SCA. The diagnosis of TTC was defined through clinical consensus based on fulfilling the following criteria: 1) An acute cardiac event typically presenting with chest pain and/or dyspnea; 2) Transient systolic dysfunction with marked left ventricular contraction abnormality (akinesia or dyskinesia of the left ventricle apical and/or midventricular or basal segments) extending beyond a single coronary perfusion bed; 3) Absence of significant (>50%) obstructive coronary artery disease or angiographic evidence of acute plaque rupture; 4) new electrocardiographic abnormalities (either ST elevation or T-wave inversion) or modest elevation in cardiac troponin level; 5) absence of pheochromocytoma; and 6) absence of myocarditis or typical ischemic trans-mural late gadolinium enhancement or Cardiac Magnetic Resonance (CMR), if available. These criteria are part of the proposed Mayo criteria for diagnosis of TTC [28].

We choose acutely decompensated chronic heart failure (CHF) patients as a control group because heart failure and TTC may share the same patho-physiological mechanism of SNS activation. Furthermore CHF is a condition known to be associated with high sympathetic tone [29] [30]. Control patients were matched for age, gender, left ventricular ejection fraction (LVEF), creatinine clearance and haemoglobinaemia, which are all factors known to interfere with MSNA activity. Creatinine clearance was assessed by Modification of Diet in Renal Disease (MDRD) formula.

The research protocol complies with the Declaration of Helsinki and was approved by the Toulouse University Hospital Human Research and Ethics Committee. Informed written consent was obtained from all control participants.

### Clinical Assessment

At admission, all patients underwent a diagnostic workup evaluation including serial electrocardiograms, blood sample analysis including cardiac enzymes, coronary angiogram ventriculogram, trans-thoracic echocardiography (TTE) with measurement of all conventional parameters and CMR imaging if possible.

### Sympathetic Nervous System Determination

Within 72 hours after the onset of symptoms, all patients (TTC and CHF control patients) underwent quantitative assessment of MSNA by microneurography. During microneurographic recording blood pressure (BP), heart rate (HR), respiratory rate and oxygen saturation were concomitantly assessed.

Microneurography was performed in the supine position, under carefully standardized conditions, in a dedicated laboratory. The laboratory is quiet, light and temperature were controlled. Since microneurography cannot be performed in the intensive care unit because of electrical interferences the only exclusion criterion was the impossibility for patients to be moved to the microneurography laboratory within 72 hours since Hospital admission.

MSNA allows a direct and dynamic evaluation of postganglionic SNS activity. As previously described [31], [32] MSNA was recorded by a tungsten microelectrode (shaft diameter 200 μm) inserted selectively in sympathetic efferent fibers journeying around the peroneal nerve. A subcutaneous reference electrode was inserted 2–3 cm away from the recording microlectrode. Once positioned, the recording microelectrode allows the collection of the electrical activity of sympathetic contingent which appears as a sequence of electrical bursts. In the absence of sensory stimuli and muscle movement the potential difference measured between the two microelectrodes is the sum of the electrical activity of orthosympathetic fibers for of the peripheral muscles vessels. The neural signals were amplified, filtered, rectified, and integrated in order to obtain a neurogramme identifying trains of SNS discharge visualized as a sequence of bursts. SNS activity can then be expressed as bursts/min and bursts/100 heart beats (hb). Heart rate was measured continuously by electrocardiogram (AD Instruments, Castle Hill, New South Wales, Australia). Blood pressure was measured continuously using the Finometer system (Finapress Medical System BV, Amsterdam, The Netherlands). Oxygen saturation was monitored with a pulse oxymeter (AD Instruments, Castle Hill, New South Wales, Australia) and respiratory rate was assessed continuously with a thoracic belt (Pneumotrace II, UFI, California, USA).

#### Spontaneous baroreflex control of sympathetic activity determination

**Figure 1:**
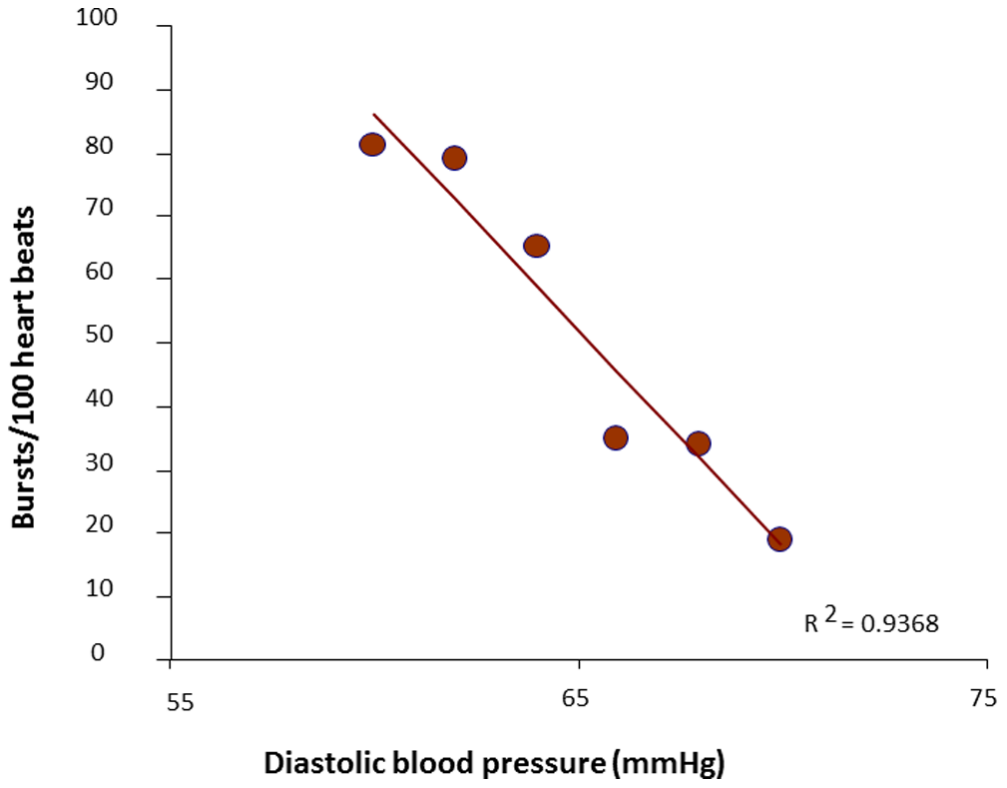
Example of a spontaneous baroreflex control of sympathetic activity determination. In this figure there is an example, in one chronic heart failure patient, of the regression line describing the relationship between Muscle Sympathetic Nervous Activity (MSNA) expressed as burst/100 heart beats (on ordinates) and concomitants spontaneous diastolic blood pressure values (on abscissae). The spontaneous baroreflex control of sympathetic activity was defined as the absolute value of this regression line.

The spontaneous baroreflex control of sympathetic activity was calculated on the basis of the microneurographic recording and concomitant blood pressure values. Assessment of spontaneous baroreflex control of sympathetic activity was performed as described previously [32]. Briefly, over a 3 to 5 min resting period, diastolic blood pressures values of individual heart beats were grouped in intervals of 2 mmHg and, for each interval, the percentage of diastoles associated with a sympathetic burst was plotted against the mean of the pressure interval (threshold diagram). Muscle sympathetic bursts were advanced by 1.3 s to compensate for baroreflex delay. The spontaneous baroreflex control of sympathetic activity was defined as the absolute value of the slope of the regression linerepresenting the relationship between MSNA (on the ordinate axis) and concomitant spontaneous diastolic blood pressure values (on the horizontal axis). In figure 1 an example, in one CHF patient, of the regression line representing the relationship between MSNA and concomitant spontaneous diastolic blood pressure values. The slope of this regression line represents the spontaneous baroreflex control of sympathetic activity value in this single patient.

### Follow-up

In-hospital clinical, biological and TTE follow-up were performed before discharge. One-year follow-up was active and was obtained in all survivors by medical visit or direct contact with their cardiologist. All events and values were prospectively site recorded.

### Statistical Analysis

The sample size has been calculated in order to demonstrate a significant difference (30%) in the primary endpoint (MSNA) between TTC patients and CHF control patients. The calculation has been made with a statistical power of 80%, a type I error rate of 5% for a two-tailed test. The sample size resulted of 12 patients.

Demographic data and baseline characteristics of the two groups (TTC patients and matched controls) were compared using a Mann and Whitney test (non parametric, unpaired test).

For every patient MSNA recording was individually examined, bursts were identified and sympathetic activity was calculated as bursts per minute and bursts for 100 heart beats (which allows comparison of sympathetic discharge between individuals). MSNA-related data were collected by FD, AV and ML, sampled by a research assistant and analyzed blindly by investigators. Measurements of MSNA were compared between the two groups using the Mann and Whitney test (non parametric unpaired test). Sympathetic baroreflex relations were analyzed by weighted linear regression (by number of beats in each diastolic range). Testing was two-sided and results are presented as median and interquartile range. Differences in categorical variables (family history of cardiovascular diseases, dyslipidemia, diabetes, etc.) have been assessed by a Fisher test.

Statistically significant differences are reported for p<0.05. Statistical analyses were performed with Graphpad Prism 5.04 (La Jolla, USA).

## Results

13 consecutive patients with a diagnosis of TTC were included. All patients were female. 13 CHF control patients were matched for age, gender, LVEF, Body Mass Index (BMI), creatinine clearance and haemoglobinaemia. Demographic characteristics of the population are depicted in Table 1. Age, LVEF, cardiovascular risk factor distribution and treatment were similar in both groups. Symptoms at presentation of TTC were: thoracic pain (6 patients, 46%), epigastric pain (2, 15%), dyspnea (4, 31%), atrial fibrillation (3, 23%), vagal symptoms (2, 15%), syncope (1 patient), shock (1 patient) and dizziness (1 patient). Nine of the 13 TTC (69%) patients reported a stressful event less than 48 hours before presentation. Triggering conditions were emotional stress in 4 patients and physical stress in 5 patients. In 4 patients (30%) any triggering event was identified. At admission 3 patients were in the New York Heart Association (NYHA) class IV (23%), 6 patients in class III (46%) and 4 patients in class II (31%). Among the 13 TTC patients, 9 had a previous psychiatric event (4 had a history of depression (31%) and 5 (38%) reported anxiety disorders). The cause of heart failure among the 13 CHF patients was ischemic coronary artery disease in 9 cases, valvular cardiopathy in 3 cases and idiopathic dilated cardiomyopathy in 1 case. The cause of acute cardiac decompensation was pneumonia in 2 cases, bronchitis in one case, a new cardiac ischemic event in 2 cases, a major physical effort in one case and in 7 patients there was any identifiable cause of cardiac decompensation. Symptoms at presentation of acute cardiac decompensation in CHF patients were: thoracic pain (2 patients, 15%), dyspnea (12, 92%), cardiogenic shock (1 patient) and nausea (1 patient). At presentation of acute cardiac decompensation, NYHA class was III in 10 patients (77%) and IV in 3 patients (23%). Among these 13 patients, 8 had a previous psychiatric event: 2 (15%) had a history of depression, 3 (23%) had a history of anxiety disorder and 3 (23%) reported both depression and anxiety disorders.

**Table 1:** Baseline characteristics of the two groups of patients.

Measurements	TTC patients (n = 13)	Control patients (n = 13)	*p*
Age, years	79 (72–88)	73 (63–85)	0.1618
Body mass index (BMI), kg/m^2^	22.2 (21.3–24.0)	24.8 (23.4–27.3)	0.0595
Coronary risk factor, n (%):			
Hypertension	9 (69%)	6 (46%)	0.2509
Hyperlipidemia	6 (46%)	8 (62%)	0.4517
Diabetes	3 (23%)	4 (31%)	0.6736
Smoking	3 (23%)	3 (23%)	1
Overweight (BMI >25 kg/m^2^)	3 (23%)	5 (38%)	0.4158
LVEF, %	40 (35–45)	27 (25–45)	0.1228.
Creatinine clearance (mL/min/1.73 m^2^)	70.6 (56.1–73.3)	51.2 (42.3–60.5)	0.1228
Hemoglobin (g/dL)	12.3 (11.4–12.7)	12.1 (11.3–13.1)	0.9603
Plasma brain natriuretic peptide, (pg/mL)	470.0 (321.1–727.7)	622.4 (405.0–1011.0)	0.3532
Treatments at explorations time, n (%)			
β-blockers	10 (76.9)	9 (69.2)	0.6736
ACEi+AT1 receptor blockers	11 (84.6)	10 (76.9)	0.6353
Diuretics	4 (30.8)	9 (69.2)	0.0524
Digoxin	0 (0)	1 (7.7)	0.3273
Vasodilators	0 (0)	2 (15.1)	0.1527
Anxiolytics	3 (23.1)	6 (46.2)	0.2326
Antidepressive agents	2 (15.3)	5 (38.4)	0.1993

LVEF (as assessed by echocardiography); ACEi: Angiotensin converting enzyme inhibitors; AT1: Angiotensin II type 1. Values are median (interquartile range).

The only exclusion criterion was then the impossibility for patients of being moved to microneurography laboratory, within 72 hours from Hospital admission, due to their critic conditions requiring permanence in an intensive care unit (ICU). Among the 13 TTC patients enrolled only 3 patients required initial admission to an ICU but the 3 of them were transferred to a traditional cardiology unit within 72 hours after admission and microneurography was performed. Among 13 acutely decompensated CHF patients, 4 were initially admitted to an ICU but transferred to a traditional care unit within 72 hours.

Values of blood pressure, heart rate, MSNA and spontaneous baroreflex control of sympathetic activity for each TTC patient are depicted in Table 2.

**Table 2:** Individual values of systolic blood pressure (SBP), diastolic blood pressure (DBP), heart rate (HR), MSNA activity and **spontaneous baroreflex control of MSNA** for the 13 Tako-Tsubo patients.

TTC patient No.	SBP	DBP	HR	MSNA	MSNA	Spontaneous baroreflex control of MSNA
	(mmHg)	(mmHg)	(hb/min)	(bursts/min)	(bursts/100 heart beats)	(%burst/mmHg)
1.	105	53	64.2	62.3	97.1	0.32
2.	114	49	64.6	55.5	85.9	0.46
3.	120	60	67.3	61.7	91.6	0.11
4.	127	64	96.5	88.7	91.9	1.29
5.	108	58	70.8	63.3	89.5	3.36
6.	91	56	66.1	65.3	98.8	0.67
7.	144	64	95.6	81. 7	85.4	1.94
8.	123	46	62.3	66	105.9	2.55
9	95	65	71.6	66	92.2	0.63
10.	142	94	67	61.3	91.6	2.39
11.	138	54	56	60.3	108	1.09
12.	152	77	54.2	73.3	135	0.35
13.	122	52	81.1	56.6	69.9	0.41

**Figure 2:**
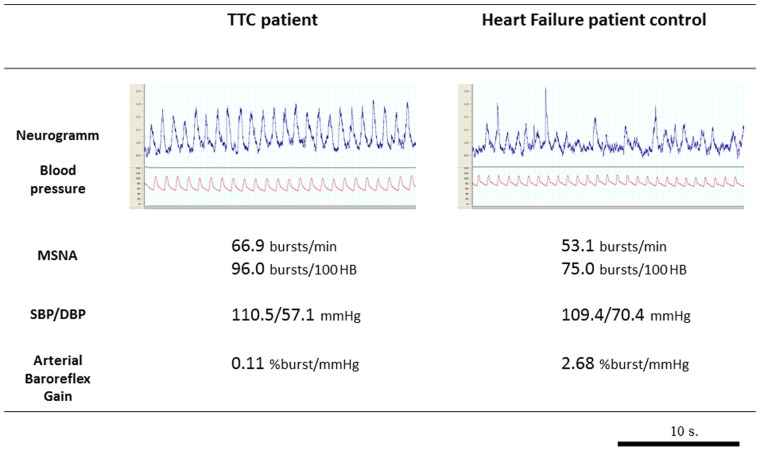
Comparison between a Tako Tsubo patient and a heart failure control patient. In this figure are showed: microneurographic recording, Muscle Sympathetic Nerve Activity (MSNA) values, Systolic and Diastolic Blood Pressure (SBP, DBP) values and spontaneous baroreflex control of sympathetic activity values in one Tako-Tsubo patient (on the left) and one chronic heart failure patient (on the right). The microneurographic recording well show the increased frequency of sympathetic bursts and then sympathetic activity in Tako-Tsubo patient compared to control.

**Figure 3:**
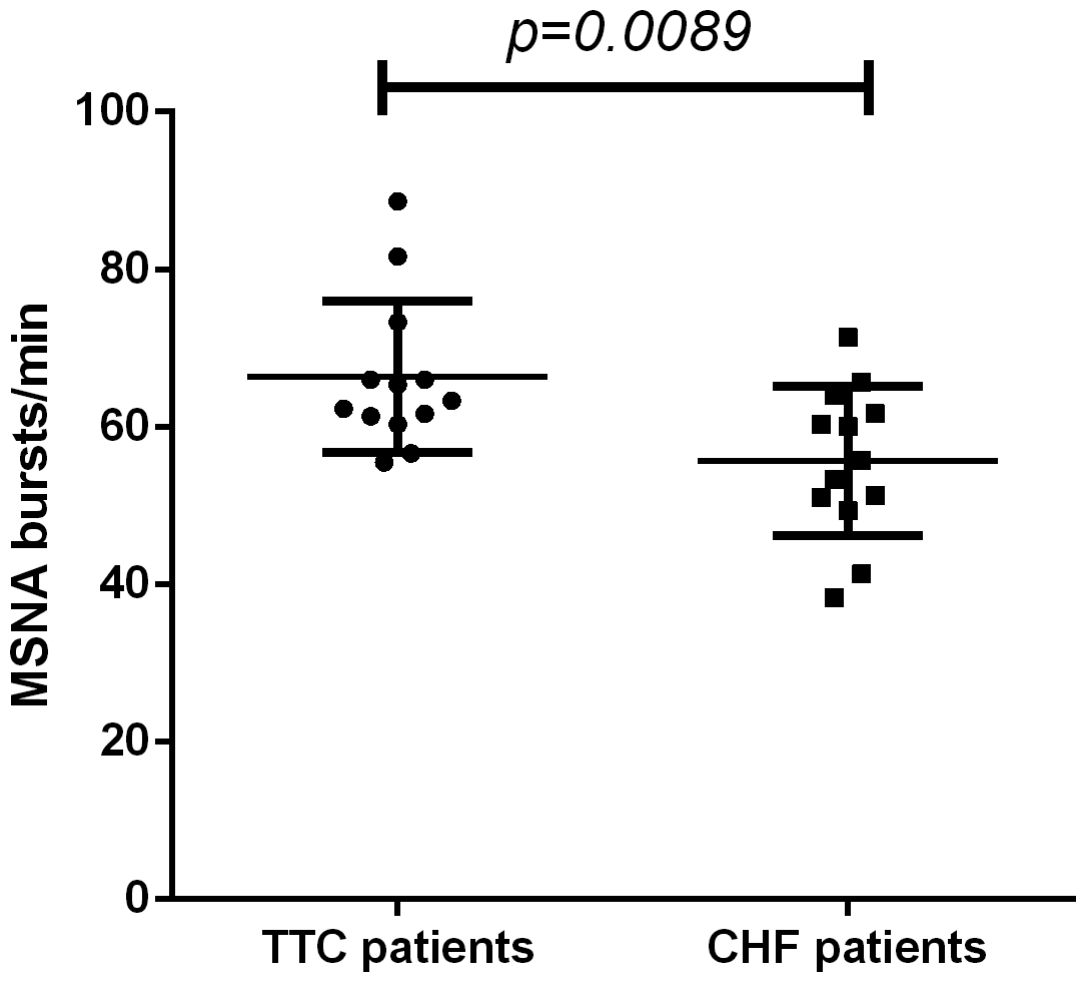
Comparison of MSNA activity between Tako Tsubo patients and CHF control patient. In this figure we show the comparison of mean MSNA activity, expressed in burst/min, between 13 TTC patients (on the left) and 13 acutely decompensated CHF patients (on the right).

Values of blood pressure, heart rate, MSNA and spontaneous baroreflex control of sympathetic activity in the two group of patients (TTC and CHF) are depicted in table 3. Between the two groups of patients there were no statistically significant differences in the values of BP, HR and oxygen saturation. TTC patients, when compared to acute decompensated CHF control patients, presented a significant increase in SNS activity assessed by MSNA and expressed as bursts/min and as bursts/100 hb, with the activity being approximately 1 burst for 1 heart beat (MSNA median 63.3 bursts/min [interquartile range 61.3 to 66.0] vs median 55.7 bursts/min [interquartile range 51.0 to 61.7]; MSNA median 91.9 bursts/100 heart beats [interquartile range 89.5 to 98.8] vs median 73.0 bursts/100 heart beats [interquartile range 68.9 to 82.2]) (figure 2 and figure 3).

**Table 3:** Baseline values of the two groups of patients.

Measurements	TTC patients (n = 13)	Heart Failure controls patients (n = 13)	*p*
Systolic Blood Pressure, mmHg	121.9 (107.6–138.2)	108.1 (96.1–121.1)	0.1627
Diastolic Blood Pressure, mmHg	58.3 (52.8–64.3)	53.3 (41.8–60.5)	0.133
Mean Blood Pressure, mmHg	78.4 (72.6–83.7)	72.4 (64.1–79.9)	0.1074
Heart Rate, beat/min	67.0 (64.2–71.6)	73.2 (63.2–82.2)	0.4684
Oxygen saturation, %	95.5 (91.2–96.7)	95.6 (93.6–96.4)	0.7088
MSNA, bursts/min	63.3 (61.3–66.0)	55.7 (51.0–61.7)	0.0089
MSNA, bursts/100 heart beats	91.9 (89.5–98.8)	73.0 (68.9–82.2)	0.0012
**Spontaneous baroreflex control of MSNA**, %burst/mmHg	0.7 (0.4–1.9)	2.4 (1.8–2.9)	0.005

Values are median (interquartile range).

**Figure 4:**
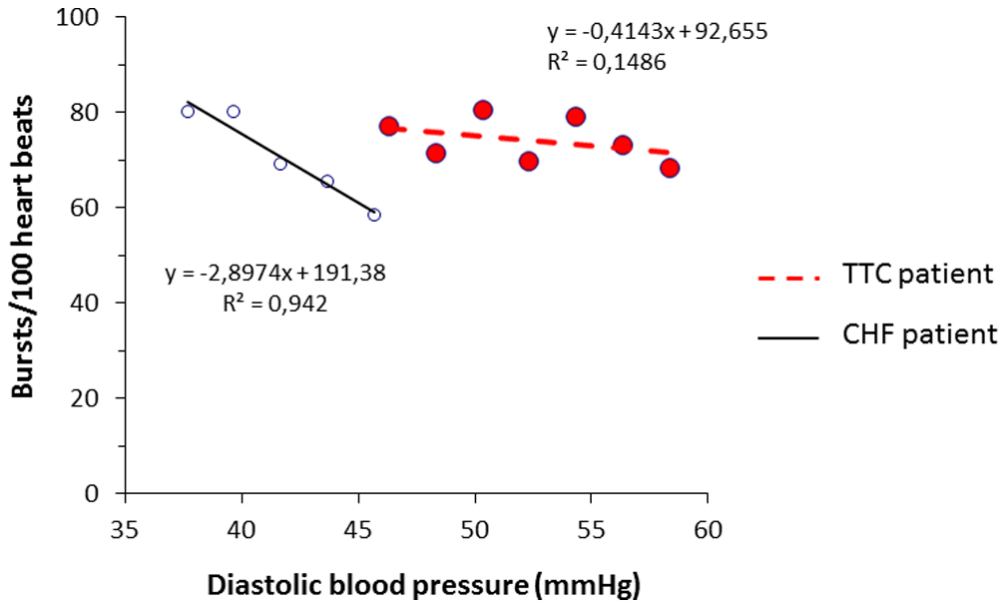
Comparison of the spontaneous baroreflex control of sympathetic activity between TTC and CHF patients. In this figure we show the comparison of the spontaneous baroreflex control of sympathetic activity of a single TTC patient (on the right) and a single CHF control patient (on the left).

TTC patients when compared to CHF control patients presented a significant decrease in spontaneous baroreflex control of sympathetic activity (table 3 and figure 4).

A significant correlation was observed between MSNA and creatinine clearance (r^2^ = 0.0394, slope = −0.2828, p = 0.0394) and between MSNA and heart rate (r^2^ = 0.4058, slope = 0.4639, p = 0.0192).

None of the TTC patients and of the control patients died during hospitalization. At 1-year follow up only one TTC patient died because of a pre-existing colon cancer with liver metastases, and 2 CHF patients died (one because of a septic shock and the other because of a pulmonary embolism).

At hospital discharge all Tako Tsubo patients presented improvement of LVEF which reverted to normality. At one year NYHA class was II for 4 patients and I for 8 patients. No recurrence of Tako Tsubo syndrome has been observed at one year among the twelve survived patients.

## Discussion

Our study not only directly confirms SNS hyperactivation in TTC patients but also provide direct evidence of the presence of an impaired spontaneous baroreflex control of sympathetic activity in these patients. The evidence of baroreflex impairment in TTC offers a possible contribution to the comprehension of TCC physiopathology.

Despite the selection of CHF controls, who are already known to have elevated SNS activity [30], TTC patients have an even higher baseline activity than patients with severe CHF. It is usually stated, that the MSNA burst rate cannot exceed heart rate but in our case-control study some TTC patients had more sympathetic bursts than QRS complexes within a time period. This observation suggests that TTC patients are characterized by a specific new pattern of autonomic dysfunction. This could be explained by the loss of baroreflex inhibitory action on SNS activity as showed in this study. Our study points to a link between sympathetic baroreflex failure and TTC, and highlights the important role of sympathetic discharge in the pathophysiology of TTC.

In our study population 31% of the TTC patients had in their past medical history previous major depressive episodes and 38% presented generalized anxiety trouble. The mechanism of sympatho-excitation in patients with depressive or anxiety disorder remains uncertain but an autonomic dysregulation able to increase sympathetic nerve activity and leading to left ventricular dysfunction has been proposed [33], [34]. Using direct cardiac catheterization techniques coupled with NE isotope dilution methodology we showed that whole body and cardiac sympathetic nervous activity in patients with depression follows a bimodal distribution, with values in some patients (around 30%) being extraordinarily high [35]. Interestingly, depressed patients also presented with a defect in function of the NE transporter. Reuptake of NE into sympathetic nerves after its release terminates the neural signal. A fault in transmitter inactivation may augment the effects of sympathetic nerve traffic. In the healthy heart over 80% of released NE is recaptured into sympathetic nerves, so the heart is more sensitive than all other organs to impairments in transmitter reuptake. Through causing persistence of the sympathetic neurotransmitter in the synaptic space, and consequently augmenting the sympathetic neural signal, such an abnormality may be an important causal factor in generating the cardiac presentation of TTC.

While the principal neuronal circuit involved in the reflex regulation of the cardiovascular system resides in the medulla, reciprocal connections between the medulla, pons, midbrain and hypothalamus are essential for the integration of behaviorally significant responses [36]. Indeed, central noradrenergic, serotonergic and amino acid neuronal pathways dysfunction has been demonstrated in patients with left ventricular dysfunction [37]. Moreover, in the largest series described by Yoshimura et al. TTC occurred in 1.2% of ischemic stroke patients. The majority of the TTC patients documented by Yoshimura et al. had stroke in the insula or close to it [38]. These data underline the pathophysiological concept of a centrally autonomic-mediated origin of TTC, because the insular cortex is regarded to contribute to cortical control of autonomic cardiac function [39].

Our data confirm that TTC occurs mainly in post-menopausal women. In fact, aging and the pre-monopausal to post-menopausal transition are associated with decrease of baroreflex sensitivity [40] and sympathetic activation. In addition, cardiovascular β-adrenoreceptor responsiveness is decreased and α1-adrenoreceptor responsiveness is increased in postmenopausal women. Therefore, sympathetic dominance replaces parasympathetic one as the main regulator of the cardiovascular system in postmenopausal women. These changes would affect cardiovascular responses during acute stress, including heart rate increases and vasoconstriction, and might help explain the increased incidence of TTC in these patients.

Studies exploring pathophysiological features of TTC mainly converge towards a common pathway, i.e. namely sympathetic nervous system activation. However, up to now, all previous studies only collected indirect measurement of SNS activity and none has been able to provide data about baroreflex function in TTC patients. Plasma catecholamine levels at presentation are usually markedly higher among patients with stress-induced cardiomyopathy. This is particularly the case for catecholamines, their precursor and neuronal metabolites, during acute phase (between day 1 and 5). We hypothesized that elevated nerve firing, as assessed by microneurography in our study, coupled possibly with impaired neuronal NE reuptake, is probably responsible for the known high catecholamine levels observed in these patients.

Finally, our results indicate that elevated sympathetic activity during the acute phase of TTC is associated with a marked alteration in sympathetic baroreflex function favoring substantially greater response in sympathetic nerve firing to spontaneous fluctuation in diastolic blood pressure. It has been suggested in a case report that afferent baroreflex failure could be associated to TTC [27], [41]. In this previously published case-report, lesions of both solitary tract nuclei, where impulses arising from baroreceptors converge, preceded TTC. However the patient had disseminated encephalomyelitis associated to this brainstem involvement and thus the causality relationship is questionable. Our study provides the first evidence that TTC is effectively associated to a decrease of the spontaneous baroreflex control of sympathetic activity.

### Study Limitations

Our work does not indicate whether the excessive sympathetic activation in TTC is limited only to the muscular vasoconstrictor bed or whether it is generalized to the whole cardiovascular system. For example, heart rate is not increased in TTC patients despite sympathetic activation. This could be related to modification of the beta adrenergic pathway [17] or abnormalities in intracellular Ca^2+^ regulation [42] seen in TTC patients. On the other side, various studies showed that in TTC sympathetic outflow is increased at the peripheral [18], [43]–[45] and cardiac level [19]. Moreover, several studies have shown a powerful correlation between MSNA and cardiac sympathetic firing [26] [46] particularly in the setting of left ventricular dysfunction. Cardiac SNS activity can be assessed by NE spill over, but unfortunately this technique was not available in our investigation center.

We have only used heart failure patient as control patient, because there are known to have elevated SNS activity. Our hypothesis was that if Takotsubo patient have a higher SNS activity than patient know to have the most elevated activity then our study would be clinically relevant. However from a pathophysiological perspective having a “clinically matched” group of female subject with acute coronary syndrome, or a “real” control group of age matched healthy female subjects without cardiac disease could have been interesting. However data from the literature show that these type of patients have a lover SNS activity than heart failure patient. Hence using these type of control patient would have facilitated positive results.

In this study we did not analyze baroreflex control of MSNA after pharmacological intervention. It appears complicated to use this technique in patients with low ejection fraction since drugs delivered for that purpose impair the hemodynamic status and can perhaps interact antagonistically with patient treatment. Moreover, there are several limitations in the interpretation of sympathetic response to vasoactive drugs, among them nitroprusside inhibition of sympathetic neurotransmission [47], or unpredictable effects of cardiac loading conditions on low-pressure mechanoreceptor nerve firing.

We have not investigated other reflexes known to increase sympathetic outflow. Metabolic and mechanical stimuli from the contracting muscles can lead to an increase in SNS activity by taking a route which bypasses the classical afferent baroreflex pathway and does not involve the solitary tract nuclei in the brainstem. This mechanism has been suggested in a case-report of post physical exercise TTC [27]. However in our study, physical exercise has not been identified as a trigger factor leading to TTC. Moreover mechanoreflex analysis implies the use of a handgrip test, difficult to perform in our cohort of very elderly patients and poorly reproducible. Activation of peripheral chemoreceptors leads to sympathetic activation [32], but in the present study, factors known to increase peripheral chemoreceptor activity in CHF patients such as creatinine clearance [32], baseline oxygen saturation and hemoglobin levels [48] were similar in both groups. We have previously shown that chemoreflex activation can contribute to sympathetic baroreflex impairment [32]. Hence it cannot be excluded that autonomic dysfunction in TTC could be mediated by peripheral chemoreflex activation.

The effect of the treatment on SNS activity is also a possible limitation of our study. For ethical and clinical reasons treatment was not withdrawn. However the percentage of patients treated with β-blockers and renin-angiotensin-aldosterone-system blockers was not significantly different between the two groups.

### Conclusion

The present study, using direct sympathetic nerve recording, demonstrates that in TTC patients sympathetic activity is increased in the acute phase and it is associated with impairment in spontaneous baroreflex control of sympathetic activity. These observations provide a contribution to the comprehension of the pathophysiology of autonomic failure in TTC. Altogether these data may be of potential relevance for possible future treatment of TTC and for improving outcome. More generally our study underlines the need to pursue further investigation about the role of the brain-heart axis in stress cardiomyopathies and in heart failure pathophysiology.
